# Cystic Tumor of the Neck Treated with Ergot

**Published:** 1878-06-20

**Authors:** B. M. Walker

**Affiliations:** Va.


### SOUTHERN MEDICAL RECORD.

VOL. VIII. ATLANTA, GA., JUNE 20, 1878. NUMBER 6.

EDITORS:

THOMAS S. POWELL, M. D. W. T. GOLDSMITH, M. D. R. C. WORD, M. D.

*R. C. WORD, M. D., Managing Editor.*

SUBSCRIPTION—$2.00 PER ANNUM, IN ADVANCE.

☞ All Communications and Letters on Business connected with THE RECORD, for 1877 and 187, must be addressed to the Managing Editor.

### *ORIGINAL AND SELECTED.*

### CYSTIC TUMOR OF THE NECK TREATED WITH ERGOT.

By B. M. WALKER, M.D., of Va.

On the 10th of February last, I was called to see Mrs. W—— with a tumor upon her neck; and as the enlargement was rapidly increasing, her mental anxiety was considerably exercised. There was no pain, only an uncomfortable feeling, and when I pressed upon the enlargement, she experienced an asthmatic sensation. On examination, I found a cystic tumor, the size of a hen's egg, occupying the front portion of the neck, between the hyoid bone and the sternum, deep in the cellular substance, between the sterno-hyoid and sterno-thyroid muscles. Fluctuation was only moderately perceptible by careful manipulation. There was firm attachment of the posterior wall of the tumor to the trachea, as evidenced in trying to enucleate it with the fingers, and better still, by its corresponding motions upwards and downwards as the trachea rose or fell. Modern surgeons, than whom none stand higher than Professor Gross, say that excision is the treatment.

I called my patient's attention—and also her husband's—to this method of cure. They seemed willing. But as it was an operation of which they had some fears, they requested me first to write their parents in New York. This I did, and sent a carefully prepared account of it to their family physician there, who agreed that it was a case for the knife. I then suggested a visit to New York for them, but they desired the operation performed here, only making one request—i. e., that I should take two months to use any rational medicinal treatment to effect a cure. The patient, being a lady twenty-three years of age, feared the scar, I think, as much as she did the operation. I consented to use my skill in this way: began giving her Squibb's fluid extract ergot, 15 gtts. in syrup sarsaparilla three times daily. At first, I determined to try the injection of Bonjean's ergotine, hypodermically, in the manner laid down by Professor Esmarch in a paper read before the fourth congress of the German Surgical Society, of Berlin, in which he treats deep-seated astheromatous cysts of the neck, first, with one per cent. sol. carbol. acid, which washed out the cyst; then he used from ten to twenty grammes of Lugol's iodine solution. Had I failed in the treatment above given, I would have given this high authority proper consideration, and then used iodine in stead of ergot—though I must admit that the course is not divested of serious risk, where such violent inflammations are set up adjacent to, and relatively connected with, the most important structures of the economy.

After persistent treatment with ergot for four weeks, occasionally using the external application of tinct. iodine, I could discover scarcely any appreciable change. The family maintained that none was apparent. Early, however, in the fifth week, we marked a decided difference in size, which was soon one-half, and a change in texture also. It had now become quite hard and more sensitive.

In addition to my treatment, I combined the iodide of potash, in 5-grain doses, three times daily, and in ten days the whole structure had disappeared.

In the use of the ergot, I could but observe its wonderful anti-phlogistic properties in controlling fever. My attention was first directed to this in reading a lecture delivered by Professor Jacobi, April 15, 1870, at the clinic of the College of Physicians and Surgeons, New York.

My patient suffering at the time of the first administration of the ergot with periodical fever, I clearly observed the controlling influence it had on the fever, always lowering the temperature and allaying nervous action.

Brown-Sequard maintains that it acts through the nervous system, and especially through the sympathetic upon the unstriped muscular tissue under its control. Thus it produces its effects upon the uterus, bladder, and the muscular coats of the arteries, diminishing the supply, and where there is a foreign element, so to speak. As tumors are not supplied with a vis a tergo of circulation, and in some instances draw their nutrition from a very meager source, may we not—reasoning *a priori*—cut off this supply—strangulate, as it were, this abnormal growth, and when we have diminished its nutrition, with evidence of its diminishing proportions, then stimulate the absorbents with that most wonderful of all medicines—iodide of potassium—to remove for us what remains? This seems to me the rational treatment, particularly when the life of the growth approaches the cystic form of disease.

Again, I cannot too strongly suggest the trial to those who have not already done so, of ergot in the treatment of their intermittent and remittent fevers, where we always find more or less nervous disturbance; acting, as it does, through the nervous system, it holds an inhibitory influence over this morbid nervous action.

One or two remarks upon ergotism of the older writers, which I believe to be a phantom of the present day. The lamented Anstie long since declared it a bug-bear. I don't believe that the ergotism, as described by older writers, either the spasmodic or gangrenous, pertains to a population like ours. Theirs was a population half-starved compared to ours, where their only food was rye, and the rye was almost exclusively ergot. Then they had nothing to supply their wants, and ergotism was admissible. Not so in a country as varied in its bountiful supplies of meats (flesh of all kinds), breadstuffs too numerous to mention, with heaven's bountiful supply of fresh milk and butter. Here ergotism will not be feared.

